# Unmanned Aircraft Systems for Studying Spatial Abundance of Ungulates: Relevance to Spatial Epidemiology

**DOI:** 10.1371/journal.pone.0115608

**Published:** 2014-12-31

**Authors:** José A. Barasona, Margarita Mulero-Pázmány, Pelayo Acevedo, Juan J. Negro, María J. Torres, Christian Gortázar, Joaquín Vicente

**Affiliations:** 1 SaBio IREC, National Wildlife Research Institute (CSIC-UCLM-JCCM), Ciudad Real, Spain; 2 Department of Evolutionary Ecology, Doñana Biological Station, CSIC, Seville, Spain; 3 Department of Microbiology, Universidad de Sevilla, Seville, Spain; INIAV, I.P.- National Institute of Agriculture and Veterinary Research, Portugal

## Abstract

Complex ecological and epidemiological systems require multidisciplinary and innovative research. Low cost unmanned aircraft systems (UAS) can provide information on the spatial pattern of hosts’ distribution and abundance, which is crucial as regards modelling the determinants of disease transmission and persistence on a fine spatial scale. In this context we have studied the spatial epidemiology of tuberculosis (TB) in the ungulate community of Doñana National Park (South-western Spain) by modelling species host (red deer, fallow deer and cattle) abundance at fine spatial scale. The use of UAS high-resolution images has allowed us to collect data to model the environmental determinants of host abundance, and in a further step to evaluate their relationships with the spatial risk of TB throughout the ungulate community. We discuss the ecological, epidemiological and logistic conditions under which UAS may contribute to study the wildlife/livestock sanitary interface, where the spatial aggregation of hosts becomes crucial. These findings are relevant for planning and implementing research, fundamentally when managing disease in multi-host systems, and focusing on risky areas. Therefore, managers should prioritize the implementation of control strategies to reduce disease of conservation, economic and social relevance.

## Introduction

Understanding the spatial distribution of risks for multi-host diseases in complex ecological scenarios is a key issue for disease management in wildlife [1] and also at the domestic/wildlife interface [2]. Population demography, resource allocation and behaviour determine the spatiotemporal structure of interactions among hosts. These characteristics may therefore play an important role in pathogen transmission [3], [4], [5]. The distribution range and abundance of domestic and wild animals can be affected by natural (biotic and abiotic) and human-mediated factors [6]. In an epidemiological context these factors are able to drive intra and inter-specific disease transmission rates [7]. For instance, areas commonly grazed by both cattle and wildlife - including artificial feeding and watering areas - have been proved to increase the risk of pathogen transmission between species [8], [9]. But risk factor analyses are highly dependent on the spatial scale, as the relationships between factors and the transmission rates may change with the selection of different areal units [10].

Determining the patterns of host abundance at a finer resolution for large areas requires an impressive sampling effort and/or the use of predictive modelling (e.g. [11], [12]). The use of expensive methods, such as manned aircrafts, may apply to wildlife monitoring [13]. Low cost unmanned aircraft systems (hereafter UAS) have recently emerged as an efficient alternative for wildlife monitoring and ecological research [13], [14]. These systems offer the ability to monitor biological processes of large areas remotely and rapidly. Thus, UAS may become an affordable, safe and accurate option for a wide variety of studies [15], [16], [17]. UAS equipped with on board cameras have been used to obtain high-resolution images of wildlife occurrences in quasi real time in highly dynamic landscapes (e.g. [16], [18]), but their possible efficacy in predicting distribution and abundance of animals has not been proven. UAS therefore have a high potential as regards collecting precise information on hosts, which is highly prized by epidemiologists since it is useful to determine the spatial risk of disease transmission (e.g. [19]).

Most multi-host diseases (e.g. foot and mouth disease, rabies, anthrax, brucellosis and tuberculosis) often emerge and/or persist in complex ecological communities involving several domestic and wild hosts [20], [21]. Of special relevance are diseases involving wild ungulates in their epidemiological cycles, as these species are undergoing a generalised expansion (e.g. [22]). This promotes epidemiological scenarios in which disease transmission often results in the maintenance or amplification of diseases that affect not only wild ungulates, but also livestock production, public health and endangered wildlife species (e.g. [23], [24]). This research proposes an epidemiological approach to evaluate applications of UAS as feasible tool for monitoring wildlife distribution and abundance.

Tuberculosis (TB), which is caused by the *Mycobacterium tuberculosis* complex, is an important re-emerging zoonotic disease that is shared between cattle and wildlife, and the existence of wildlife reservoirs limits the control of this disease [21], [25]. The TB infection generally develops into chronic infections, with long-term persistence in populations, and is able to induce a period of infectiousness during which direct and indirect contact favouring disease transmission between individuals (intra and inter-specific) occurs. In South-central Spain, complex epidemiological connections between TB prevalence in cattle and wild ungulates (wild boar *Sus scrofa*, red deer *Cervus elaphus* and fallow deer *Dama dama*) have been established [23], [26], thus highlighting the role of wildlife as TB maintenance hosts in this complex system (see also [8], [23], [27]).

Since environmental features (e.g. natural water sources, water holes, marsh-shrub ecotone and/or grazing areas) frequently used by hosts could act as important sources of TB and/or favour closer contact of individuals both between and within the species, we hypothesise that the pattern of TB frequency can be explained by the spatial patterns of the host abundance. Our aim when using UAS high-resolution images was specifically to: i) model the spatial pattern of abundance for each potential host species in a Mediterranean scenario, and ii) evaluate the explanatory capacity of the predicted abundance of hosts with the spatial risk of TB throughout the ungulate community as a way to validate the predictions of host abundance obtained from UAS data.

## Materials and Methods

### Ethics statement

This study utilized samples from wild ungulates captured (shot by the Park’s rangers) and necropsied in the context of the Doñana National Park health-monitoring programme. Sampling followed a protocol approved by the Animal Experiment Committee of Castilla-La Mancha University and by the Spanish Ethics Committee, and designed and developed by scientists (B and C animal experimentation categories) in accordance with EC Directive 86/609/EEC for animal handling and experiments. The work was conducted complying with the current Spanish legislation involving aviation safety and field technicians had the required licenses to operate in the frequencies they used. Doñana National Park authorities (P43-2010), MINECO (AGL2010-20730-C02-01) and Aeromab Project (P07-RNM-03246) field permits approved these methods.

### Study area

**Figure 1:**
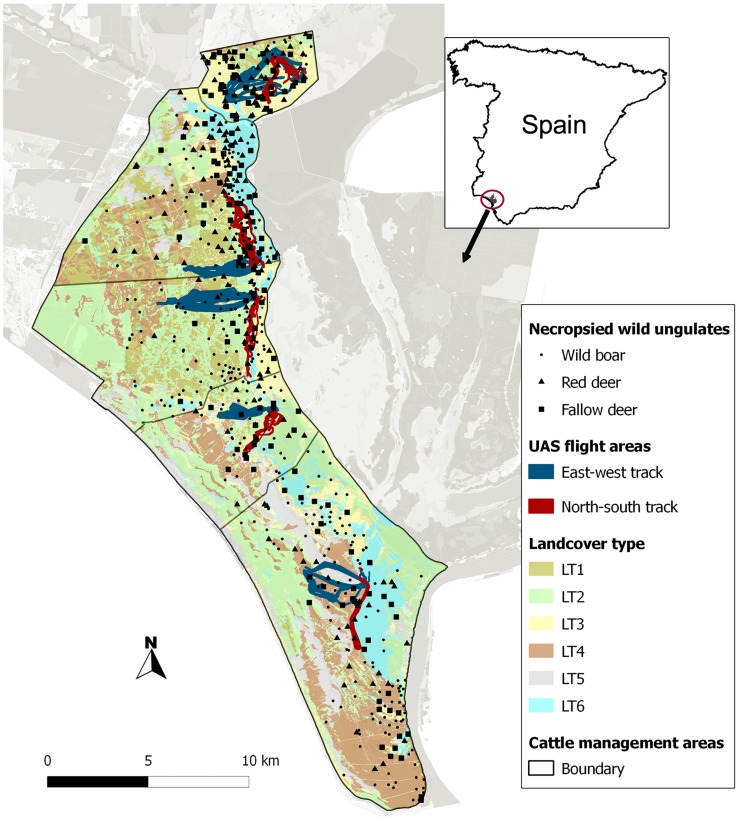
Map of the study area, Doñana National Park in Southern Spain, obtained from [36]. Six main habitats can be differentiated: dense shrubland (LT1), low-clear shrubland (LT2), herbaceous grassland (LT3), woodland (LT4), bare land (LT5), watercourse vegetation and water body (LT6). Locations of the necropsied wild ungulates and of the UAS tracks at the five cattle management areas are shown.

The study was carried out in Doñana National Park, DNP (37°0′ N, 6°30′ W, 54,000 ha), a nature reserve located on the Atlantic coast of South-western Spain. DNP has the highest level of environmental protection in Spain and is one of the most important natural reserves in Europe in terms of biodiversity. The region has a dry sub-humid Mediterranean climate with marked seasons. In the wet season (winter and spring), the marshlands are flooded and ungulates graze in the more elevated shrublands. The hardest season for ungulates in this area is summer (from July to September), when herbaceous vegetation, wetlands and water bodies (i.e., there is no water flow during this season) dry up in most habitats, and only a few meadows remain green on the boundary between the upper area of shrubs and the low dry marsh (ecotone). During the dry season, a north-south oriented longitudinal humid ecotone – dominated by wetland plants including *Scirpus maritimus* and *Galio palustris* with *Juncus maritimus* associations – can be identified between the shrublands and the edge of the dry marshlands (Fig. 1).

A traditional breed of cattle (locally called “marismeña”) is present in DNP, in which 5 different cattle management areas (MA) can be identified from north to south (Fig. 1). The southern MA, Marismillas (MA1, n = 318 cattle) has a density of 3.1 cattle/km^2^. The central area comprises 3 MA (MA2, n = 152 cattle; MA3, n = 168 cattle and MA4, n = 350 cattle) and an average of density of 4.2 cattle/km^2^. There are no cattle in the northern MA, which is called Coto del Rey (MA5). Wild ungulates in the area include: wild boar, red deer and fallow deer. Agriculture and hunting are prohibited in DNP, and artificial feeding practices are not conducted. However, cattle TB reactor rates are still high in DNP despite compulsory testing and the culling of infected animals [23], [28]. Previous studies have revealed that *M. bovis* infection prevalence is spatially structured, leading to among the highest rates of TB reported in wildlife worldwide [23], [27]. In addition to behavioural factors, it is probable that environmental features may explain variations in TB rates in wildlife since they favour direct and/or indirect contact between individuals and species during the dry season [8], [29].

### Unmanned Aircraft Systems (UAS) methodology

We performed UAS fieldwork during the summer (August and September) of 2011, the season when water resources are more limited for wildlife, the availability of food is severely reduced [30] and the aggregation of individuals around water resources is therefore expected to be at its maximum [31]. The UAS platform was built using the foam fuselage of a radio controlled model Easy Fly plane (St-models, China) propelled by a brushless electrical engine. The embarked systems are: an on-board video camera used for First Person View Flight (FPV), a GPS (10 Hz, Mediatek, model FGPMMOPA6B), an Ikarus autopilot (Electronica RC, Spain) which provides flight stabilization, On Screen Display (OSD), a Panasonic Lumix LX-3 digital photo camera 11MP (Osaka, Japan), a three dimension waypoint following capability and an “emergency return home” function. The ground station is composed of a case containing a monitor, a DVD recorder, a video receiver and the control signal transmitter with the corresponding antennas. It also includes a Laptop with which to program the autopilot, store the pictures and data logs, and decode in-flight telemetry, thus allowing the position of the UAS to be tracked in real time on a Microsoft map (Redmond, WA, USA). For further details please see [17] and [18].

We conducted a total of 60 aerial tracks (each of which was ∼4 km in length and 0.1 km wide: total surveyed length 240 km, covering 10.1% of the study area) between 17.30 h and 21.00 h local time, the time when the animals are most active as the temperature begins to decrease [32]. Two types of tracks – east-west and north-south oriented transects (Fig. 1) – were surveyed in each MA and replicated six times. The UAS were programmed to fly at an altitude of 100 m and at an average speed of 40 km/h. Details of the image processing can be found in [18]. The information obtained was divided into patches of 1 ha (our territorial unit for modelling). The patch size was adjusted to the width (0.1 km) of aerial tracks which is also consistent with a previous study on deer spatial ecology in the study area [33]. For each territorial unit and species, the abundance index (our response variables for modelling) was estimated as the number of individuals seen per ha.

### Environmental predictors for modelling host abundance

Environmental variables were chosen on the basis of their availability for the study area and their potential predictive power (e.g. [14], [31], [34]). The predictors calculated for each territorial unit were: straight-line distance (km) to nearest artificial water hole (DW); straight-line distance (km) to nearest marsh–shrub ecotone (DE); exacted grid area (ha) surveyed by UAS (GA); number of water points per km^2^ (WDN); proportion of dense scrub (LT1); proportion of low-clear shrubland (LT2); proportion of herbaceous grassland (LT3); proportion of woodland (LT4); proportion of bare land (LT5); and proportion of watercourse vegetation (LT6; summarized in Table 1). Landcover data was obtained from Andalusia Environmental Information [35].

**Table 1:** Environmental predictors used for spatial modelling of both host species abundance and *Mycobacterium tuberculosis* complex transmission risk factors.

Codes	Descriptions	Mean	SD
DW	Distance to nearest water point (km)	**0.45**/0.97	**0.36**/0.71
DE	Distance to nearest marsh-shrub ecotone (km)	**1.01**/2.33	**0.89**/2.15
GA	UAS grid area (ha)	**1.23**/1	**0.80**/0.00
WDN	Water point density (n/km^2^)	**0.82**/0.51	**1.18**/0.35
LT1	Dense shrub (%)	**20.94**/11.39	**34.47**/26.51
LT2	Low-clear shrub (%)	**23.72**/30.56	**33.08**/39.41
LT3	Herbaceous grassland (%)	**24.91**/11.55	**36.99**/30.78
LT4	Woodland (%)	**14.66**/17.69	**31.43**/34.04
LT5	Bare land (%)	**7.12**/10.64	**21.83**/26.11
LT6	Water course vegetation (%)	**5.87**/10.92	**19.25**/27.84
MA	Cattle management area (factor, 5 categories)	**–**	**–**

For continuous predictors, mean values and standard deviations (SD) are reported for both the surveyed area (UAS track grids; in bold) and the whole study area (surveyed/study area).

Canopy cover reduces visibility and thus the detection of ungulates in UAS images. We therefore calculated detection coefficients in order to take this fact into consideration. Detection coefficients were calculated for both types of vegetation cover, dense shrub land (LT1) and woodland (LT4), in order to correct the reduction of visibility when ungulates were found below these canopy covers. We analysed the probability of detection of 100 random circle points (1 m^2^ size) per habitat image (surface = 1 ha), considering any point above vegetation cover as “not detected” and any point without vegetation cover as “detected”. This was repeated in ten different images of each cattle management area and landcover type (n = 100 images). For each landcover type, the detection coefficient is calculated as the proportion of points that were detected per image. The detection coefficients used in the statistical analyses were 0.538 and 0.359 as thresholds of visibility in LT1 and LT4 cover types, respectively. This means that we could only detect 53.8% of the ungulates located in LT1 habitat and 35.9% of the ungulates located in LT4. Collinear variables were excluded using a variance inflation factor (VIF) coefficient>3 threshold cut-off value [36].

### Modelling ungulate abundance

Since information on ungulate distribution for the whole DNP was incomplete, we used spatially explicit modelling for predicting abundance values for overall study area. Habitat-species relationships can be parameterised with this feasible and reliable tool from the sampling obtained with UAS. The statistical model obtained could then be used to forecast species abundance in areas where information about the target species was not available [12]. When calibrating the abundance model, we only used the east-west UAS track data because we found very low habitat variation in the north-south UAS tracks (mainly overlapping with ecotone habitat), whereas the east-west tracks randomly sample the different habitats present in DNP. Previously to modelling, the dataset from the east-west UAS was randomly split by using a subset to parameterise the models (70%), whereas the rest (30%) was reserved for model validation on independent data. The north-south UAS track data were, however, also used to validate predictions from modelling using another set of independent data.

For each species (red deer, fallow deer and cattle - wild boar were excluded owing to low diurnal detection of the species; see [37]), the response variable was the abundance index for the territorial units. Then, the values of each environmental predictor were summarized for each sampling unit using ‘zonal statistic tool’ with Quantum GIS version 1.8.0 Lisboa [38]. The local abundance of each species per territorial unit was modelled using a generalised linear model, with a negative binomial distribution and a logarithmic link function [39]. We opted for the negative binomial distribution owing to high levels of overdispersion in the data when the models were fitted with Poisson distributions. The final models were obtained using a forward-backward stepwise procedure based on Akaike Information Criteria (AIC; [40]).

After modelling, predicted abundances were quantitatively compared with the data observed in the validation datasets by using Pearson’s correlations (significant threshold; *p*<0.05) on both the east-west UAS independent data and the north-south UAS data. All statistics were carried out using R 2.15.2 [41].

### Sampling and tuberculosis diagnosis

From 2006 to 2012, 949 wild ungulates comprising wild boar (n = 570), red deer (n = 190) and fallow deer (n = 189) were randomly captured, necropsied and sampled in the context of the DNP health-monitoring programme (Fig. 1). The exact shooting location of each animal sampled was geo-referenced with a portable GPS (GarminLtd., Cayman Islands). Species, sampling year, gender and age were recorded on each of the animals sampled using similar criteria to previous studies (see [23]). Briefly, age classes were typified as juveniles (<24 months old) or adults (>24 months old) [8]. Necropsies were performed by qualified wildlife veterinarians with a wide experience in the diagnostics of macroscopic TB compatible lesions via the detailed inspection of lymph nodes and abdominal and thoracic organs (see [42]). Cultures were performed in order to confirm TB, using Lowenstein-Jensen piruvate medium. In the four MA in which cattle were present, veterinarian authorities used skin tests on the cattle populations and positive reactors were slaughtered during same period, as part of the National TB control Programme.

### Determining the risk of TB at spatial scale

**Figure 2:**
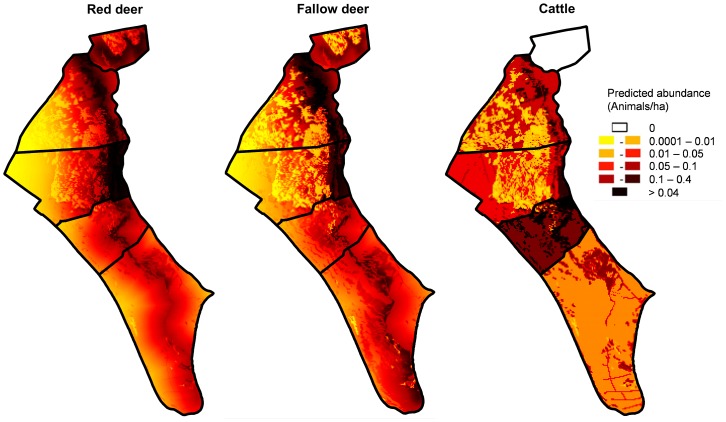
Predicted patterns of species relative abundance (animals/ha) in Doñana National Park obtained by modelling abundance data obtained from Unmanned Aircraft Systems (see **Table 2**).

We used a Bayesian multivariable logistic regression approach [43] to evaluate the association among the occurrence of TB at individual level (response variable) and the predicted species abundances, while adjusting for the other risk factors hypothesised to influence disease status in DNP [26]. Predicted abundances of each modelled species (obtained in the UAS approach at 1 ha resolution; Fig. 2) were assigned to each TB-tested animal by using ‘point sampling tool’ with Quantum GIS. In addition, the covariates related to aggregation risky points (i.e., DW and WDN, see Table 1), species, sex, age class and sampling year of TB-tested animals were also included in the model (S1 File). In Bayesian inference, uncertainty in estimating predictor is fully reflected in the estimates and credible intervals (CIs) of all other variables, which is particularly important when there are a number of explanatory variables and smooth terms available [44]. This Bayesian framework was run using Integrated Nested Laplace Approximations (R-INLA package; [45]) to avoid the convergence and mixing algorithms of the Markov Chain Monte Carlo based sampling methods [46]. Deviance information criterion (DIC) was used for Bayesian model selection by following a backward stepwise procedure and the model with the lowest value of DIC was retained [47]. The statistical significance of each explanatory variable was assessed by 95% CIs of the probability distribution.

## Results

### Spatial distribution of ungulates abundance

The aerial tracks allowed a total of 3,149 ungulates to be located, identified and recorded from the UAS images. This included 51.44% of red deer, 33.18% of fallow deer, 11.36% of cattle, 2.89% of horses and only 0.22% of wild boar, while 0.89% was recorded as unknown species. The results of the best-fitting abundance models and their coefficients for the environmental predictors are summarized in Table 2. The common landscape predictors affecting the abundance indices explained by the best-fitting models for all species were: LT1 (negatively related to dense shrub), LT3 (positively related to herbaceous grassland) and MA (Table 2; see for more details S1 Table).

**Table 2:** Results of the generalised linear models (negative binomial error distribution and logarithmic link function) used to predict red deer, fallow deer and cattle abundance on a spatial scale in Doñana National Park.

Response	Final model
Red deer abundance	∼ −0.001·DE −2.96·^1^LT1+0.78·LT3+0.44·^2^MA2+2.15·MA3+1.1·MA4+0.4·MA5
Fallow deer abundance	∼ −2.17·DE −2.25·^1^LT1+2.9·LT3+7.22·^1^LT4 −1.42·^2^MA2+2.73·MA3+3.68·MA4 −1.34·MA5
Cattle abundance	∼ −1.19·^1^LT1+2.93·LT3+3.36·^2^MA2+6.6·MA3+9.17·MA4 −3.41·MA5

Coefficients are shown for the most parsimonious models according to AIC. Measures for model support and statistical parameters (test and p-values) for the variables selected in the final models can be found in S1 Table. Variable codes are described in Table 1.

1 LT1 and LT4 were corrected by detection coefficients, 0.538 and 0.359, respectively. ^2^Reference value of the parameter estimator was 0 for “cattle management area 1 (MA1)”. MA from 2 to 5 codify for the location of each sampling unit in each management area (1 when present and 0 when absence).

Validation on independent datasets for both east-west and north-south UAS track data showed significant correlations between observed and predicted abundance values (Table 3). After being assessed using independent data, the final models were used to predict species abundance in the whole of DNP (Fig. 2). Common distribution patterns can be observed in the specific predictions of species abundance throughout DNP. It is remarkable that all ungulate species tended to spatially aggregate at the ecotone. Differences between MA were more evident for cattle.

**Table 3:** Assessment of the predictive performance of the abundance models in independent datasets by using Pearson correlations (*p*<0.001 in all cases).

Abundance models		
Model	East-west UAS track data (30%)	North-south UAS track data
	*Pearson’s r, n = 501 grids*	*Pearson’s r, n = 1013 grids*
Red deer	0.32	0.26
Fallow deer	0.36	0.23
Cattle	0.19	0.34

### Spatial risk factors for TB infection

Infection was detected in 55.69% (SE = 4.12%; n = 570) of wild boar, 35.79% (SE = 6.86%; n = 190) of red deer and 17.99% (SE = 5.49%; n = 189) of fallow deer. From 2006 to 2013, official skin testing of the 1,139 cattle in the DNP study area revealed a maintained mean incidence of 9.04% (SE = 4.91%) TB reactors.

Variables in the best-fitting Bayesian model included the following predictors: species sampled, WDN, predicted abundance of red deer and cattle (Table 4; the distribution probability for each parameter is shown in S1 Fig.). The Bayesian inference revealed that the increasing of TB risk was significantly associated with the high predicted abundances for red deer (estimated as 0.21; SD = 0.05) and cattle (estimated as 0.12; SD = 0.07). The infection risk was higher in wild boar than red deer, but higher in red deer than in fallow deer. In general, areas with low values of WDN were mostly related to wild ungulates infections.

**Table 4:** Results of the best-fitting (i.e. the lowest DIC value) Bayesian multivariable logistic regression model used to determine the most relevant factors explaining species positivity as regards tuberculosis.

Variable		Coefficient	SD	95% CI	
				2.5%	97.5%
Intercept		–8.17	0.19	–8.57	–7.79
Species	Red deer	0.71*	0.21	0.30	1.13
	Wild boar	1.31*	0.18	0.95	1.68
WDN		–1.50*	0.30	–2.11	–0.89
PRD		0.21*	0.05	0.09	0.31
PCT		0.12*	0.07	–0.04	0.26

Explanatory categorical variable was the TB-tested species: fallow deer, red deer and wild boar. Quantitative variables were water point density (WDN) and predicted species abundance (PRD in red deer and PCT in cattle) obtained from the models summarised in Table 2 and Fig. 2. Coefficients, standard deviations (SD) and 95% credible intervals (CI) are shown. Coefficients of species categories are relative to the fallow deer. Significant variables are marked with (*).

## Discussion

This research provides a multidisciplinary approach to study complex ecological and epidemiological systems, which may benefit from innovative technologies previously implemented in other fields, such as UAS. Although other study designs are possible, this research could be considered as a case study where UAS technology became a valuable tool to address host community abundance at finer spatial scale. Thus, UAS is a promising alternative in studies that require the estimation of spatial patterns of different species in large areas –like epidemiological ones- where traditional methods (e.g. line transects, drive counts, etc.) are not feasible due to logistical reasons. Our study identified points of high host abundance, which were associated with a high risk of TB infection, probably because these areas could act as important sources of TB and/or favour effective intra- and inter-specific contacts. Results allowed to identify the cattle/wildlife interface in which control strategies for TB – and other diseases that are modulated by similar risk factors – should be prioritised, which is particularly crucial for the development of disease control policies in the context of shared diseases.

### On the methodological approach

Remote observation technologies based on low cost UAS have the potential to be a reliable and efficient alternative to study the distribution of ungulate abundance in open landscapes where multiple wild species can be differentiated by high quality imagery. Therefore, UAS constituted an excellent tool for wildlife monitoring in general and spatial epidemiology in particular. The valuable advantages of UAS respect to other methodologies are its low impact on wildlife behaviour and its capacity to provide high spatio-temporal accurate information on the surveyed populations at open landscapes. For instance, high-resolution images obtained from UAS (S2 Fig.) allowed us to record, for each species, the local number of individuals and to avoid the animals’ reactive responses to observer presence [16]. In DNP, a relatively open study area, we successfully identified the species in 99.1% of the animals detected. This result contrasts with previous studies using UAS pictures with rapid naked-eye image analysis, that were not able to distinguish, at species level, herbivores smaller than elephants, also in open habitats [16]. Differences between studies could be related with a combination of methodological factors such as: the height of the UAS (100–750 m [16] versus 100 m in this study) and the cruise speed (an average of 80 km/h [16] versus 40 km/h in this study).

The high precision obtained with UAS and the cost-effectiveness of the method suppose a notable advantage of this system when monitoring wildlife distribution in large territories, because the effort required to obtain accurate information for large areas – as demanded by epidemiologists – is unaffordable and/or unapproachable with the use of traditional methodologies (e.g. [11]). Other cost-effective indirect indicators of species presence and/or abundance, such as droppings (e.g. [48]) and browsing indices (e.g. [49]), may not be able to distinguish between species, and therefore are not useful to study multihost systems. However, accuracy and variation in animal detection and identification from UAS images may depend, among other things, on habitat visual permeability and the species’ behavioural traits (e.g. [50]). On the one hand, habitat detectability can be taken into account to estimate corrected abundances in closed habitats, as it was done in this study. On the other, daytime elusive cryptic species such as wild boar [37] are under-represented in the surveys (compared with independent data, Natural Processes Monitoring Team at EBD, http://www-rbd.ebd.csic.es/Seguimiento/mediobiologico.htm). No information with which to model wild boar abundance was consequently obtained in this study. Future improvements to the UAS must address this by for instance incorporating systems with thermal sensors that are able to detect animals at night [17], [51].

### Spatial patterns of ungulate abundance and TB infection

We have modelled the spatial distribution of wild ungulates and cattle on a very fine-scale throughout DNP. In general, all the epidemiologically-connected species selected the ecotone. This habitat offers key resources, namely food and shelter (see also [31]), which are more relevant during the summer when drought severely reduces the availability of resources [30]. We found differences in the spatial patterns predicted for each species among the MA for all the species studied. In the case of wild ungulates, this probably resembles differences in the species’ habitat selection, as they have different ecological requirements on a local scale (e.g. [52]). In particular, fallow deer had a higher predicted abundance than red deer and cattle in the southernmost MA. Stone pine *(Pinus pinea*) reforestations perform especially well in the southernmost MA of DNP, and favour good pasture, which attracts fallow deer, which is probably the European deer species that is most prone to grazing (e.g. [22]). Differences among MA were quite significant for cattle as a consequence of the DNP cattle farming plan which compulsorily establishes the maximum cattle stocks in each MA.

The host spatial patterns were recorded in summer 2011, when the maximum aggregation of hosts is expected. However, TB data in sampled animals were collected during 2006–2012. Therefore, to study the relationship between host abundance and disease, we assumed that the spatial patterns did not change from year to year or seasons. Our design is realistic due to TB is a chronic disease and seasonal changes are not marked. Published observations on ungulate distribution confirm these seasonal patterns are consistent every year [31]. In addition, the sampling years of the sampled animals were included in the risk model, which controlled the effect of the changes in the spatial pattern. Nonetheless future studies should address how yearly changes (or due to the precipitation regime and water distribution in summer) in spatial distribution affect the TB epidemiology and its spatial pattern.

By joining abundance, environmental and disease data in a unique Bayesian modelling approach we have shown that spatial variation in the TB risk throughout DNP could relate to: (i) TB-tested species, (iii) environmental features, and/or (iii) predicted abundance of ungulates from UAS data. TB risk was higher in wild boar than red and fallow deer which is consistent with the infection pattern founded in this shared disease at South-central Spain, as has previously been demonstrated by both field and molecular epidemiology [23], [27]. The Bayesian inference revealed that the high TB risk for wild ungulates was negatively associated to water point densities. As the density of water points increases, lower levels of aggregation are expected at these points, and the risk for (direct and/or indirect) disease transmission should subsequently decrease [8], [53]. Water points and irrigated and cultivated fields have been identified as high-risk areas for disease transmission between feral pigs (*Sus scrofa*) and cattle in Mediterranean areas from USA [5]. These complex epidemiological scenarios have also been described in dry areas in Africa, in which cattle share water points and diseases with wildlife [54], [55]. In South Spain, a recent study has evidenced that wildlife-livestock interactions occur much more often at water resources than would be expected to occur by chance, and has argued that water points should be considered as potential hotspots for TB transmission between wildlife and cattle on extensive farms [53]. As a step forward, an experimental study in South Spain evidenced that effective segregation strategies of wildlife and cattle at water points under dry Mediterranean conditions have the potential to reduce inter-specific contacts and TB transmission at the wildlife/cattle interface [29]. Further research should therefore focus on the environmental persistence of *M. bovis* at watering sites in ecosystems with marked dry seasons, particularly where water becomes limited and leads to high animal aggregations.

TB risk was also positively associated with the high-predicted abundance of ungulates and consequently served as an actual way to evaluate the explanatory capacity of the predictions obtained by the UAS approach. The effect of local host abundance on the inter-specific transmission is probably environmentally mediated (indirect transmission) in Mediterranean areas [53], whereas direct interactions, especially at intra social group level, may be more relevant as regards determining direct rates for intra-species transmission [27]. Blanchong et al. [56] evidenced that direct effective contacts within white-tailed deer *Odocoileus virginianus* family groups were a significant mechanism for disease transmission. Apart from water points, other scattered environmental resources have the potential for disease transmission, such as grasslands, especially at the ecotone, where ungulates aggregate in order to forage during the summer. Future studies, which may benefit from the continued use of UAS for wildlife monitoring, should take into account the seasonality of resources in a highly variable ecosystem like DNP in order to assess both intra and inter-annual differences in the species habitat selection and thus in the risk factors that drive disease transmission.

## Supporting Information

S1 Fig **Estimated probability distribution by Bayesian modelling.** Posterior probability distribution of the variables included in the best-fitting Bayesian model to evaluate the association among the occurrence of TB at individual level and the predicted species abundances, while adjusting for the other risk factors hypothesised to influence disease status in Doñana National Park.
(DOCX)

S2 Fig **High-resolution image obtained from UAS camera.** High-resolution image obtained from Unmaned Aircraft System camera. Domestic and wild ungulates aggregated in the dry marshland of Doñana National Park are observed.
(DOCX)

S1 Table **Results of abundance models.** Results of the generalised lineal models (negative binomial error distribution and logarithmic link function) used to predict red deer, fallow deer and cattle abundance on a spatial scale in Doñana National Park. Statistical parameters, coefficients (test-value), are shown for the best-fitting models (in bold). Variable codes are described in Table 1. Measures for model support (Akaike’s information criterion; AIC and ΔAIC) are included.
(DOCX)

S1 File **Datasets of TB-tested animals and predicted abundances.**
(XLSX)
