# Supplementary material for: Unmanned Aircraft Systems for Studying Spatial Abundance of Ungulates: Relevance to Spatial Epidemiology
Source: PLoS One. 2014 Dec 31;9(12):e115608. doi: 10.1371/journal.pone.0115608 (PMC4281124; doi:10.1371/journal.pone.0115608)
Supplement: S1 Fig — Estimated probability distribution by Bayesian modelling. Posterior probability distribution of the variables included in the best-fitting Bayesian model to evaluate the association among the occurrence of TB at individual level and the predicted species abundances, while adjusting for the other risk factors hypothesised to influence disease status in Doñana National Park. (DOCX) [file pone.0115608.s001.docx]

**Supporting information**

**Figure S2. Estimated probability distribution by Bayesian modelling**

**
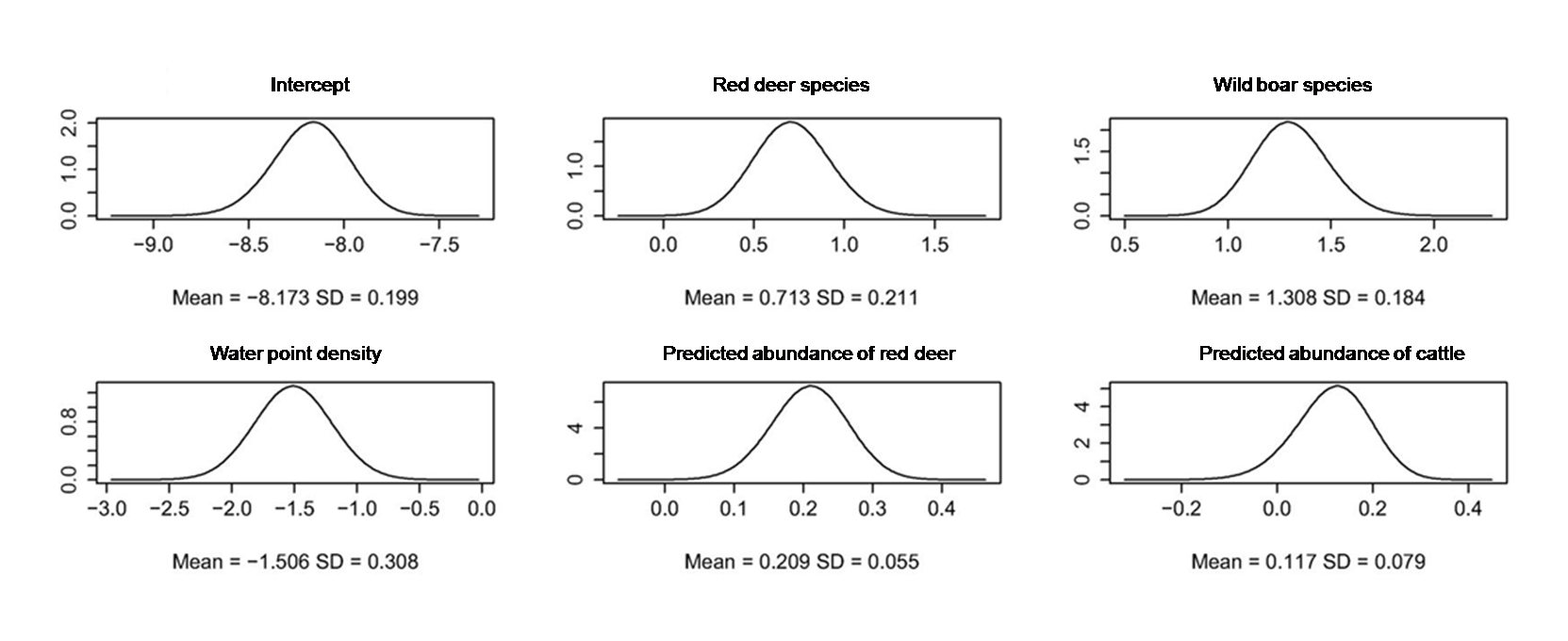
**

**Figure S2.** Posterior probability distribution of the variables included in the best-fitting Bayesian model to evaluate the association among the occurrence of TB at individual level and the predicted species abundances, while adjusting for the other risk factors hypothesised to influence disease status in Doñana National Park.
