# Supplementary material for: Unmanned Aircraft Systems for Studying Spatial Abundance of Ungulates: Relevance to Spatial Epidemiology
Source: PLoS One. 2014 Dec 31;9(12):e115608. doi: 10.1371/journal.pone.0115608 (PMC4281124; doi:10.1371/journal.pone.0115608)
Supplement: S2 Fig — High-resolution image obtained from UAS camera. High-resolution image obtained from Unmaned Aircraft System camera. Domestic and wild ungulates aggregated in the dry marshland of Doñana National Park are observed. (DOCX) [file pone.0115608.s002.docx]

**Supporting information**

**Figure S3. High-resolution image obtained from UAS camera**

**
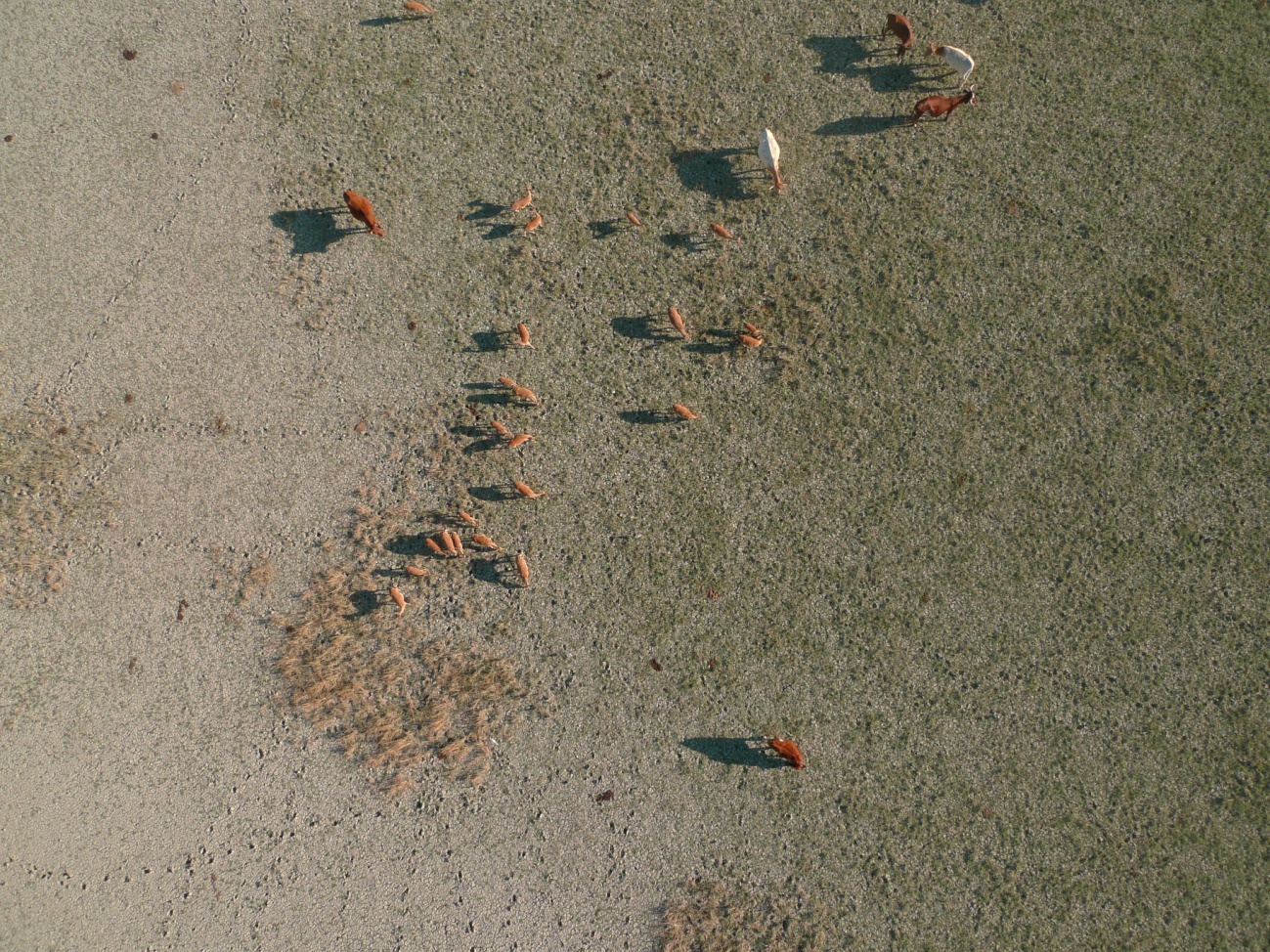
**

**Figure S3.** High-resolution image obtained from Unmaned Aircraft System camera. Domestic and wild ungulates aggregated in the dry marshland of Doñana National Park are observed.
