# Supplementary material for: Unmanned Aircraft Systems for Studying Spatial Abundance of Ungulates: Relevance to Spatial Epidemiology
Source: PLoS One. 2014 Dec 31;9(12):e115608. doi: 10.1371/journal.pone.0115608 (PMC4281124; doi:10.1371/journal.pone.0115608)
Supplement: S1 Table — Results of abundance models. Results of the generalised lineal models (negative binomial error distribution and logarithmic link function) used to predict red deer, fallow deer and cattle abundance on a spatial scale in Doñana National Park. Statistical parameters, coefficients (test-value), are shown for the best-fitting models (in bold). Variable codes are described in Table 1. Measures for model support (Akaike’s information criterion; AIC and ΔAIC) are included. (DOCX) [file pone.0115608.s003.docx]

**Supporting information**

**Table S1**. **Results of abundance models.** Results of the generalised lineal models (negative binomial error distribution and logarithmic link function) used to predict red deer, fallow deer and cattle abundance on a spatial scale in Doñana National Park. Statistical parameters, coefficients (test-value), are shown for the best-fitting models (in bold). Variable codes are described in Table 1. Measures for model support (Akaike’s information criterion; AIC and ΔAIC) are included.

| Response | Model | Coefficients (t-value) | AIC | ΔAIC |
| --- | --- | --- | --- | --- |
| Red deer abundance | Best-ﬁtting | **~ -0.001 · DE (-4.89***) -2.96 · ^1^LT1 (-3.03**) +0.78 · LT3 (1.71ns) +0.44 · ^2^MA2 (0.88ns) +2.15 · MA3 (4.8***) +1.1 · MA4 (2.06*) +0.4 · MA5 (0.7 ns)** | **1463** | **0** |
|  | Initial | ~ DW + DE + GA + **^1^**LT1 + LT2 + LT3 + **^1^**LT4 + LT5 + LT6 + MA (noted as Global) | 1538 | 75 |
| Fallow deer abundance | Best-ﬁtting | **~ -2.17 · DE (-3.85***) -2.25 · ^1^LT1 (-3.04**) + 2.9 · LT3 (3.55***) + 7.22 · ^1^LT4 (2.25*) -1.42 · ^2^MA2 (-1.31ns) +2.73 · MA3 (3.1**) +3.68 · MA4 (3.82***) –1.34 · MA5 (-1.26ns)** | **560** | **0** |
|  | Initial | Global | 653 | 93 |
|  |  |  |  |  |
| Cattle abundance | Best-ﬁtting | **~ -1.19 · ^1^LT1 (-2.67**) +2.93 · LT3 (3.9***) +3.36 · ^2^MA2 (4.14***) +6.6 · MA3 (0.78ns) +9.17 · MA4 (1.1 ns) -3.41 · MA5 (0.1ns)** | **552** | **0** |
|  | Initial | Global | 609 | 57 |

^1^LT1 and LT4 were corrected by detection coefficients, 0.538 and 0.359, respectively. ^2^Reference value of the parameter estimator was 0 for “cattle management area 1 (MA1)”.

*P* values are shown: ns *p*> 0.05, * *p*< 0.05, ** *p*< 0.01 and *** *p*< 0.001.
